# Preliminary evaluation of the BeH20 co-parenting program using a multiple baseline experimental design

**DOI:** 10.1111/fcre.70085

**Published:** 2026-07-06

**Authors:** Rebecca Hoppe, Disha Kotecha, Karey L. O’Hara, Trina Nudson

**Affiliations:** 1 Department of Psychology, Reach Institute at Arizona State University, Tempe, Arizona, USA; 2 School of Interdisciplinary Forensics, Arizona State University, Glendale, Arizona, USA; 3 The Layne Project, Inc., Olathe, Kansas, USA

**Keywords:** co-parenting, divorce, high conflict parents

## Abstract

This study evaluated BeH20, a community-embedded co-parenting program for divorced and separated parents experiencing high interparental conflict, using two complementary designs. In Study 1, 102 parents completed a one-time post-only survey, rating perceived changes in their co-parenting quality, program components, overall satisfaction, and likelihood of recommending BeH20. Results showed a large, significant improvement in perceived co-parenting quality, strong agreement that the program offered practical communication strategies, businesslike conflict-resolution techniques, guidance on separating marital vs. co-parenting roles, and support for a child’s relationship with both parents, as well as high overall satisfaction and recommendation likelihood. In Study 2, 11 parents participated in a multiple-baseline, single-case experimental design, providing 462 daily observations of co-parenting (respect, animosity) and nonreactivity, and 519 observations of conflict tactics (constructive vs. destructive) across baseline, intervention, and follow-up. Findings revealed a significant reduction in animosity during the intervention phase. Together, these results offer preliminary evidence that BeH20 is both highly acceptable and effective at targeting a key mechanism of change—reducing animosity between co-parents—and demonstrate the feasibility of conducting rigorous, university–community evaluations of community-embedded programs. Future work should extend these partnerships to explore additional mechanisms of change and streamline data collection processes for scalable implementation.

## INTRODUCTION

Divorce and separation mark a profound shift in family structure and daily life. Each year, nearly 1,100,000 children in the United States experience parental divorce (Kreider & Ellis, 2011). Although divorce rates have stabilized, these figures likely underestimate family disruption, as they exclude separations that occur outside of the legal system (Cherlin, 2017; Kreider & Ellis, 2011). Conflict is common during separation/divorce, but ongoing high-conflict dynamics are estimated in 5% and 10% of separating and divorcing families (Neff & Cooper, 2004; Schepard, 2004). With over 4,000,000 domestic relations cases filed annually in the United States (Gibson et al., 2024), this translates to 200,000–400,000 high-conflict families affected each year. Persistent interparental conflict not only strains legal resources but also undermines efforts to protect children from both short and long-term harm.

Children who experience parental separation/divorce show greater risk across a range of adverse outcomes, including risky behaviors (Barrett & Turner, 2006), early departure from the family home (Goldscheider & Goldscheider, 1998), and internalizing (Amato, 2000, 2001), academic (Anthony et al., 2014), and social adjustment (Amato, 2001; Kunz, 2001) problems. Without appropriate interventions, risk often persists beyond the short-term, worsening over time (Cherlin et al., 1998), and cascading into other problems in adulthood such as higher rates of physical and mental health problems (Lacey et al., 2013; Tebeka et al., 2016), substance use disorder (Barrett & Turner, 2006), long-term relationship difficulties (Webster & Herzog, 1995), and lower financial and job success (Sun & Li, 2008; Zill et al., 1993).

Although separation/divorce is associated with adverse outcomes for children, decades of research have shown that these effects vary considerably across families (Amato & Anthony, 2014; Amato & Keith, 1991; Hetherington & Kelly, 2002). In fact, most children of separated/divorced parents adapt to their new family structure and circumstances, going on to lead healthy, functional lives. Still, a sizable portion of children experience greater emotional and behavioral difficulties than their peers residing with cohabitating or married parents.

The leading empirical and theoretically supported risk factor for maladjustment is the stress associated with the separation/divorce (Wolchik et al., 2023). Parental separation/divorce represents a major life transition for the whole family and is often accompanied by increased exposure to interparental conflict (IPC) and disruptions in parenting, residence, and school. Decades of research show that children exposed to IPC are at greatest risk for long-term mental health challenges (Amato & Keith, 1991, Buehler et al., 1997; Harold & Sellers, 2018; van Eldik et al., 2020). IPC is a wide-ranging set of parent behaviors that can be categorized as constructive or destructive based on its frequency, severity, content, and resolution (Harold et al., 2016; O’Hara et al., 2022). O’Hara et al. (2019) found that 27% of children who reported frequent and intense exposure to IPC had a diagnosable mental health disorder 6–8 years post-divorce, nearly double the 13% observed in low-IPC divorced families. Notably, even when IPC diminished over time, children initially exposed to high levels of conflict continued to show elevated risk, underscoring the lasting impact of early IPC exposure.

IPC is theorized to impact children’s mental health directly and indirectly. According to emotional security theory (EST, Cummings & Davies, 2010), destructive IPC, characterized by animosity, coerciveness, aggression, and escalating anger, undermines children’s sense of safety and security in their family relationships. Children who witness frequent, hostile exchanges between parents come to view their family as unstable and threatening, eroding their emotional security and psychological stability (Davies & Cummings, 1994). Multiple studies have shown that IPC predicts greater emotional insecurity, and in turn, is associated with elevated internalizing and externalizing symptoms, for children in married (Cummings et al., 2012, 2015; Cummings & Miller-Graff, 2015; Davies, Hentges, et al., 2016; Davies, Martin, et al., 2016; de Silva et al., 2021) and divorced families (Asanjarani et al., 2021; O’Hara et al., 2021, 2023).

Additionally, the Spillover Hypothesis suggests that IPC negatively affects parenting, leading to reduced warmth, greater irritability, and inconsistent discipline, which further impairs children’s adjustment (Erel & Burman, 1995; Krishnakumar & Buehler, 2000). In a meta-analysis, van Dijk et al. (2020) found that IPC predicted child adjustment problems through disruptions in parenting. Specifically, lower levels of parental support and structure, along with higher levels of hostility, intrusiveness, and role confusion, significantly mediated the link between IPC and both internalizing and externalizing symptoms. While most of these associations were small, they were statistically robust and consistent across studies.

While conflict is inevitable, its impact on child development depends on its nature (Cummings & Davies, 2010; Goeke-Morey et al., 2007; Sandler et al., 2008). Destructive IPC is consistently associated with poor child outcomes (Grych & Fincham, 2001), but constructive IPC, characterized by a calm, respectful, and solution-focused perspective, can be protective, contributing to lower levels of internalizing and externalizing problems in children (Goeke-Morey et al., 2007; Sandler et al., 2008). Therefore, parents need not eliminate conflict entirely but promote constructive behaviors to buffer children from negative post-divorce consequences (Brock & Kochanska, 2015; Cheung et al., 2016; Cummings et al., 2003).

Parents’ constructive behavior forms the foundation for constructive conflict. Mindful parents tend to be more emotionally regulated, less reactive, and better able to remain present during stressful moments (Duncan et al., 2009). Mutual respect between co-parents fosters cooperation and helps maintain calm, composed communication, even in disagreement (Hastings and Grusec 1998). The effects of constructive IPC extend into parent–child interactions (Cummings et al., 2004; Rinaldi & Howe, 2003), by positively influencing parenting practices, promoting warmth, consistency, and reducing psychological control between parents and children (Duncan et al., 2009). Parents who use calm reasoning and respectful communication with one another are more likely to use similar strategies with their children (Warmuth et al., 2020). Conversely, those who engage in hostile communication or emotional withdrawal with a partner often carry those patterns into parenting (Rinaldi & Howe, 2003). Sherrill et al. (2017) found that constructive conflict with a partner on a given day predicts more constructive parenting that day and vice versa, showing a dynamic bidirectional effect.

There is also growing evidence that constructive IPC helps shield children from negative emotional outcomes. Children who witness respectful and supportive conflict resolution between parents are less likely to intervene in the conflict and more likely to maintain a sense of emotional security (Cummings & Davies, 1996). Lee et al. (2019) found that maternal reports of constructive conflict were associated with lower child emotional insecurity and behavior problems, suggesting that *how* parents argue matters more than *whether* or *how often* they argue. Thus, equipping parents with constructive conflict skills offers a promising way to mitigate IPC’s negative impacts and foster resilience in their children.

### Parenting interventions to reduce risk factors and promote protective factors

A growing body of parenting interventions, grounded in developmental and behavioral theory, demonstrates that reducing destructive IPC and strengthening parenting practices substantially lower children’s risk for adverse outcomes after separation or divorce (Braver et al., 2005; Carr et al., 2017; Wolchik et al., 2007). These programs draw on two complementary models. The person-environment transactional framework (Sameroff, 1975, 2000) posits that parenting and child behavior shape each other in a continuous feedback loop across development. The risk and protective factor model (Institute of Medicine, 1994) highlights how exposure to adversities (e.g., IPC, instability) and access to safeguards (e.g., nurturing caregiving, stable routines) jointly determine children’s mental-health trajectories. Together, these frameworks underscore that replacing destructive conflict with respectful interactions and consistent caregiving not only supports emotional regulation and resilience, but also initiates a positive cycle that strengthens family dynamics over time.

The new beginnings program (NBP; Wolchik et al., 2007), rooted in these two models of change, has been shown to strengthen parent–child relationships through quality time, active listening, and effective discipline, while reducing IPC through anger management and structured communication strategies. Across multiple longitudinal randomized trials, NBP has shown sustained improvements in parenting practices and reductions in conflict, which mediate long-term gains in children’s mental health and social functioning (Wolchik et al., 1993, 2000, 2013). Dads for life (DFL; Braver & Griffin, 2000) applies similar behavioral principles, targeting divorced nonresident fathers to promote emotional regulation and problem-solving, strengthen father-child bonds, and reduce IPC. Like NBP, DFL explicitly teaches parenting skills such as clear and empathetic dialogue, giving affirming feedback, and respectful boundary setting, while tailoring its approach to the unique challenges of non-custodial parenting. A randomized trial of DFL showed increased paternal engagement, reduced IPC, and improved child adjustment, contributing to the growing evidence on the effectiveness of targeted interventions in improving outcomes for children in high-risk contexts (Braver et al., 2005).

The Parents Plus (PP, Carr et al., 2017) program, which has been delivered to separated/divorcing parents, has similar goals but takes a social learning theory (Bandura, 1977) and solution-focused systemic therapy (Franklin et al., 2012) driven approach. The social learning theory in this context theorizes that parents can learn both problematic and beneficial behaviors through observations, modeling, and feedback. Parents are shown vignettes depicting parenting strategies, which they practice in a guided setting, and receive feedback to reinforce the skills. Similarly, the solution-focused systemic therapy focuses on an individual to drive change by building goal-focused, collaborative, and practical solutions rather than analyzing the issues (Boscolo & Bertrando, 1996). It views the family as a system, where leveraging a parents’ existing strengths, past successes, and “what’s working” in the family can help improve communication, emotional regulation, and conflict resolution. PP has consistently demonstrated its effectiveness in enhancing parenting skills, strengthening parent–child relationships, and reducing behavioral difficulties in children across developmental stages (Carr et al., 2017).

Together, these interventions illustrate the powerful harmony between theory, practice, and outcomes. By targeting modifiable family processes, such as conflict behaviors, positive parenting practices, family dynamics, and emotional communication, these interventions offer scalable pathways to reduce risk, enhance protection, and promote positive developmental trajectories in children experiencing parental separation/divorce.

### BeH20

Much of the existing research is on interventions developed and tested in university-led trials before being implemented in community settings. Far less is known about programs that originated within communities and developed outside of controlled research environments. BeH20 is a community-embedded, 16-week intervention designed to reduce IPC and promote cooperative co-parenting through a structured combination of individual and joint (with co-parent) coaching sessions, as well as psychoeducational and support-focused group meetings. Parents may engage with BeH20 voluntarily or through court mandate. Grounded in the principle that sustained parental conflict can undermine children’s emotional security and well-being, the BeH20 curriculum encourages parents to “be water” by responding to co-parenting disagreements with calmness and flexibility rather than “fan the flames” by escalating conflict with animosity and anger. Across weekly meetings, parents learn critical skills to manage their emotions, strengthen communication and problem-solving, and develop insight into their own behavior patterns and parenting values. BeH20 culminates in the creation of a co-parenting commitment, which is shared with their child and symbolizes the progress parents have made toward building a more respectful and child-focused co-parenting relationship.

Although BeH20 is rooted in behavioral science and trauma-informed principles, its efficacy has not yet been systematically evaluated. This lack of evidence represents a critical gap in our understanding of whether and how court-affiliated community programs like BeH20 can contribute to healthier family dynamics. The present study seeks to address this gap through a university-community collaboration to evaluate program effects of BeH20.

### Current study

We evaluated BeH20 using two complementary designs to assess its (1) perceived impact and acceptability, and (2) change in theorized intervention targets. For Study 1 (Feedback Survey), 102 court-referred separated/divorced parents completed a one-time post-only survey immediately after finishing BeH20 (December 2022–May 2025). Parents characterized their co-parenting before and after BeH20, rated program components (communication strategies; separating marital vs. co-parenting roles; businesslike conflict resolution; supporting the child’s relationship with both parents), overall satisfaction, and likelihood of recommending BeH20.

For Study 2 (Pilot Study), we used a single-case experimental design to test whether BeH20 helps court-referred separated/divorced parents reduce destructive interparental conflict and improve communication and co-parenting. Across baseline, intervention, and follow-up phases, we measured animosity, respect, nonreactivity, and constructive vs. destructive conflict behaviors. We also examined how these patterns varied depending on whether participants reported contact with the other parent.

## METHOD

### Study design

This evaluation of BeH20 was conducted in partnership between a university and community-embedded organization (i.e., community partner). Participants reported on both their own and their co-parent’s behaviors, allowing assessment of co-parenting dynamics from one partner’s perspective. Although collecting data from both parents would further strengthen validity, prior research using similar diary methods has shown robust interparental agreement on conflict behaviors (Cummings, et al., 2003), supporting the reliability of single-reporter data in this context. Since Study 1 aimed to assess basic program satisfaction, we analyzed feedback from post-program surveys completed after participants’ final session. The sample included all parents who participated in BeH20 between December 2022 and May 2025 to maximize generalizability. Since Study 2 focused on preliminary target engagement, specifically, whether the program influenced key theorized change mechanisms (e.g., animosity, hostility, conflict behaviors), we conducted a pilot study using a single-case experimental design with repeated measures to examine changes in parental conflict behaviors and co-parenting dynamics over time. Using a sample independent from Study 1, this design allowed for detection of effects with a small sample size and provided a rigorous approach to control for threats to internal validity (e.g., history, repeated assessment, regression to the mean; Barlow et al., 2009; Kazdin, 2011). In the single-case experimental design, each participant’s baseline phase is used as their control condition, making it particularly effective for testing intervention outcomes while maintaining a high degree of experimental control. This is accomplished by staggering the introduction of the intervention across participants. By varying the duration of each participant’s baseline phase before beginning the intervention (e.g., introducing the intervention after five baseline observations for one subset and after seven for another), the design reduces the likelihood that observed changes are due to external events or maturation effects rather than the intervention itself. This approach strengthens internal validity and enables the establishment of a causal link between the independent and dependent variables.

### Procedures

Study 1’s sample was collected during routine program evaluation by the community partner. Study 2’s sample was collected as a pilot research study through the university. The university’s Institutional Review Board approved all study procedures. Participants could enroll in BeH20 voluntarily or through a court mandate related to separation or divorce. Participation in the research component remained voluntary. All first-time participants aged 18 and older who enrolled in BeH20 were invited to join the study. The inclusion criterion was fluency in English, as the program was conducted solely in English, and there were no exclusion criteria. The intervention was delivered by the community partner as part of their standard services for separating/divorcing parents, while the research study was conducted independently by the university. Parents who consented to participate were asked to complete a series of surveys distributed through REDCap, a secure, web-based platform. Invitations to complete surveys were sent by email or push notification, and participants could skip any questions they did not wish to answer. All invitations included a link to a list of participant resources.

The research protocol included three components: a pretest survey, daily diary surveys, and a posttest survey. The pretest was sent between 1 and 3 weeks before the first program session for staggered baseline assessment and took about 20 min to complete. It included an intake form for contact and demographic information, along with six questionnaires assessing risk for child maladjustment, co-parenting attitudes, conflict strategies, attachment security, emotion regulation, and children’s mental health (with age-specific versions). Participants also received brief daily diary check-in surveys for 18–21 weeks by email or app notification, starting between 1 and 3 weeks before the program for staggered baseline assessments, continuing through the 16-week program, and ending 1 week after. These daily surveys, which took about 5 min to complete, focused on emotional regulation and IPC events. After the program concluded, participants were invited to complete the posttest survey, which repeated the same measures as the pretest and included additional questions about program feedback, including acceptability, relevance, feasibility, and perceived impact. To thank parents for their time, the community partner provided e-gift cards for completing the research activities. The principal investigator provided the list of participants to the community partner so they could issue gift cards. Participants were informed in advance that they would receive a gift card, a common method to increase response rates without introducing systematic bias (Bruno et al., 2017). Parents received $50 for the pretest, $50 for the posttest, and up to $100 based on the proportion of daily surveys completed (out of 126 total opportunities). The community partner offered participation to eligible parents and did not collect, access, or manage any research data. Participation had no impact on parents’ court cases or their access to services.

### Participants

#### Study 1

One hundred two court-referred separated/divorced parents completed a survey immediately after completing BeH20 (December 2022–May 2025). One case was missing pre/post data (final *N* = 101).

#### Study 2

Eleven court-referred separated/divorced parents completed daily surveys over the 18-week study period (i.e., total possible number of observations was 1386).

### Measures

#### Study 1

##### Co-parenting relationship characterization

Parents rated their overall relationship with their co-parent prior to and since participating in BeH20 using an eight-point categorical scale (1 = *Extremely Hostile*; 2 = *Hostile*; 3 = *Moderately Angry*; 4 = *Cold*; 5 = *Avoidant*; 6 = *Civil*; 7 = *Friendly*; 8 = *Very Friendly*). Pre- and post-program labels were converted to numeric scores (1–8) and averaged into a difference score indicating change in perceived co-parenting quality.

##### Program components

Parents evaluated four core components of the program using single-item five-point Likert scales (1 = *Strongly Disagree* to 5 = *Strongly Agree*). Parents indicated the extent to which BeH20 offered ideas on how to communicate, separated the marital from the co-parenting relationship, and resolved conflict in a businesslike manner with the co-parent, and helped to understand the importance of allowing their child to love both parents. Mean scores were calculated for each item to gauge participants’ perceptions of BeH20’s usefulness.

##### Program satisfaction and recommendation likelihood

Parents provided two global evaluations on single-item five-point scales (1 = *Very Dissatisfied/Strongly Disagree*; 5 = *Very Satisfied/Strongly Agree*): overall satisfaction with and likelihood of recommending BeH20 to a friend. Mean scores summarized participants’ satisfaction and endorsement.

#### Study 2

##### Co-parenting dynamics

Parents self-reported on the Respect (six-items; e.g., “I value my children’s other parent’s parenting skills”) and Animosity (six-items; e.g., “I am hostile or bitter in my conversations with my child’s other parent”) subscales of the Coparenting Across Family Structures Scale Short Form (CoPAFS; Saini et al., 2022; *α* = 0.41 for respect; *α* = 0.57 for animosity). Parents rated their agreement with each statement on a five-point Likert scale (1 = *Strongly Agree* to 5 = *Strongly Disagree*). Two items from the Respect- and all items from the Animosity subscales were reverse scored. A total score was computed for each subscale by averaging across all items, with higher scores indicating greater respect and greater animosity.

##### Nonreactivity

Parents self-reported on the Nonreactivity subscale (six-items) of the Interpersonal Mindfulness Scale (IMS; Pratscher et al., 2019; *α* = 0.94). Parents rated how frequently each statement (e.g., “I take time to form my thoughts before speaking”) using a five-point Likert scale (1 = *Almost Never* to 5 = *Almost Always*). A total subscale score was computed by averaging across all items, with higher scores indicating greater nonreactivity.

##### Conflict tactics

Parents self-reported and informant-reported on the other parent’s Constructive (three-items, e.g., “calm discussion”) and Destructive conflict tactics (seven-items, e.g., “personal insult”) using the Categories and Continua of Destructive and Constructive Marital Conflict Tactics scale (Goeke-Morey et al., 2003). Parents selected all applicable tactics (0 = *No* and 1 = *Yes*). Total scores for constructive and destructive tactics per reporter were summed across all items, with higher scores indicating greater constructive and destructive tactics.

##### Co-parent contact

Parents were asked if they had any contact (e.g., email, text, phone, in-person) with the other parent that day (0 = *No* and 1 = *Yes*).

##### Parent’s feedback

Parents rated (seven-items) and described (three-written) their experience in BeH20 for acceptability (e.g., “I would recommend the BeH20 program”), relevance (e.g., “The BeH20 program seems applicable”), feasibility (e.g., “The things I learned in the BeH20 program seem doable”), and perceived impact (e.g., “After taking the BeH20 program, I feel empowered to protect my children from conflict”). Parents rated their agreement with each statement on a five-point Likert scale (1 = *Completely Disagree* to 5 = *Completely Agree*).

### Data analysis approach

#### Study 1

We first converted each categorical pre- and post-program rating (1 = *Extremely Hostile* to 8 = *Very Friendly*) into numeric scores and computed a difference score (Post – Pre) for each participant. We then conducted a paired-samples *t*-test on these difference scores, calculated Cohen’s *d* as the mean difference divided by its standard deviation and tabulated the proportions of participants showing negative (Δ < 0), no (Δ = 0), and positive change (Δ > 0). We computed the mean rating for each single-item measure to summarize participants’ perceptions of program content, overall satisfaction, and recommendation likelihood.

#### Study 2

We analyzed the daily time series data collected during the baseline and intervention phases using multilevel modeling. Longitudinal models were estimated using the PROC MIXED procedure in SAS with full information restricted maximum likelihood (REML) estimation. REML provides reliable estimates for small samples with missing data (McNeish, 2017). An advantage of using multilevel modeling in multiple-baseline designs is the ability to model all cases simultaneously using inferential statistics, rather than analyzing each participant individually using visual inspection (Ferron et al., 2009).

Despite the advantages, several methodological considerations are important when applying multilevel modeling to multiple-baseline data. First, these designs often involve small sample sizes. To address this, we applied the Kenward Roger correction to adjust degrees of freedom. This correction has been shown to yield unbiased estimates in small samples (McNeish, 2017). Second, the time series nature of the data introduces potential autocorrelation, as observations closer in time tend to be more strongly correlated than those further apart (e.g., level of anger reported today is more likely to resemble level of anger reported tomorrow compared to level of anger reported a week later). To address this, we specified a first-order autoregressive error structure (West & Hepworth, 1991). Third, multilevel models often assume a homogeneous error structure across participants, which may lead to biased estimates. Therefore, we allowed for the Level-1 error covariance matrix to vary across participants to model individual differences (Baek & Ferron, 2012). We initially specified all models with random effects for each participant (e.g., random intercepts and slopes) to capture between-person variability in change. If a model failed to converge, we removed the random slope for phase while retaining the fixed effect to model within-person change over time. Given the potential for delayed intervention effects as participants learned and practiced new skills, we also tested quadratic terms to capture nonlinear change across baseline and intervention periods.

Participants were instructed to complete daily assessments; however, some submitted responses for several days at once. To reduce bias associated with retrospective reporting, only data submitted on the actual day of reporting was retained; entries reported retrospectively were treated as missing. We used an intent-to-treat approach by including all participants who initiated the study. While participants were instructed to complete both pretest and posttest, only four did, and as a result, these analyses could not be conducted due to the insufficient sample size.

## RESULTS

### Study 1

#### Parent’s feedback

The paired-samples *t*-test (*N* = 101) showed significant improvement in the way parents characterized their co-parenting from pre- to post-program, *t*(100) = 11.37, *p* < 0.001, with a mean change of Δ = 1.99 (SD = 1.76) and a large effect size (Cohen’s *d* = 1.13). Three percent of parents reported a negative change, 19% reported no change, and 78% reported a positive change in their relationship ratings.

On average, parents agreed (*M* = 4.33) that BeH20 offered ideas for communicating with their co-parent and resolving conflict in a businesslike manner. Parents somewhat agreed (*M* = 3.99) that BeH20 provided guidance on separating marital and co-parenting roles, and they strongly agreed (*M* = 4.56) that BeH20 helped them to understand the importance of allowing their child to love both parents. Overall satisfaction (*M* = 4.01) and likelihood of recommending BeH20 to a friend (*M* = 4.02) were high.

### Study 2

#### Descriptive statistics

The sample included 55% mothers and 45% fathers within the following age ranges: 24–34 (36.4%), 35–44 (45.5%), and 45–54 (18.2%). Parents had an average of two children (range = one to four children) and self-identified as White (82%) or Black/African American (18%). Educational backgrounds varied: 36% had completed some college, 27% held a master’s degree, and 9% each had a GED, associate’s degree, bachelor’s degree, or doctoral degree. No parents reported completing only high school (12th grade) or earning a certificate.

#### Intervention effects

Both linear and quadratic terms were tested to capture potential linear and nonlinear changes across baseline and intervention periods. We retained linear models over quadratic models because both yielded similar results, and the quadratic terms did not provide additional interpretive value. Therefore, only linear models are reported. For co-parenting and non-reactivity, there were *n* = 462 observations (44.8% missing values), and for conflict tactics, there were *n* = 519 observations (53.2% missing values). See Table 1 and Figure 1.

##### Co-parenting

The intercept indicated relatively high levels of animosity at baseline (*b* = 3.69, SE = 0.12, *p* < 0.0001). Although there was a trend toward an immediate increase in animosity at the start of the intervention (*b* = 0.12, SE = 0.07, *p* = 0.09), this effect was not statistically significant. There was no significant linear change in animosity during the baseline phase (*p* = 0.16), and no significant difference in slope across phases (*p* = 0.39). However, the intervention phase slope was significant and negative (*b* = −0.004, SE = 0.001, *p* = 0.03), demonstrating a significant decrease in animosity over the course of the intervention.

Respect scores were moderate at baseline (*b* = 2.85, SE = 0.10, *p* < 0.0001), with no significant immediate shift at the start of the intervention (*p* = 0.63). Linear slopes during both baseline (*p* = 0.11) and intervention phases (*p* = 0.33), as well as changes in slope (*p* = 0.15), were not statistically significant, indicating no meaningful change in respect over time.

##### Nonreactivity

Baseline levels of nonreactivity were moderately high (*b* = 3.61, SE = 0.17, *p* < 0.0001), with no significant immediate shift at the start of the intervention (*p* = 0.74). Linear slopes during baseline (*p* = 0.52) and intervention phases (*p* = 0.18), as well as changes in slopes (*p* = 0.11), were not statistically significant, indicating no meaningful change over time.

##### Conflict tactics

Constructive conflict showed a significant immediate increase at the start of the intervention (*b* = 0.39, SE = 0.13, *p* = 0.003), while baseline levels (*p* = 0.75), slopes at baseline (*p* = 0.17) and intervention (*p* = 0.15), and changes in slopes (*p* = 0.21) were not significant, suggesting an abrupt improvement rather than gradual change.

Destructive conflict significantly decreased during the baseline phase (*b* = −0.02, SE = 0.01, *p* = 0.002), followed by a significant change in slope during the intervention (*b* = 0.02, SE = 0.01, *p* = 0.002). The level change between phases was marginal (*b* = 0.14, SE = 0.07, *p* = 0.06), suggesting some reduction in destructive conflict prior to the intervention.

Parents’ reports of their co-parent’s behavior showed a significant increase in the co-parent’s constructive conflict at the start of the intervention (*b* = 0.31, SE = 0.11, *p* = 0.007). No effects were significant for the co-parent’s destructive conflict.

##### Contact-controlled models

When controlling for the frequency of contact with the co-parent, both constructive and destructive conflict were positively associated with contact (*b* = 1.00, SE = 0.03, *p* < 0.0001; *b* = 0.12, SE = 0.02, *p* < 0.0001, respectively). Additionally, controlling for contact removed the previously significant effects for constructive and destructive conflict.

##### Parent’s feedback

Parents reported positive experiences with BeH20. Most described BeH20 as helpful, enjoyable, and empowering, with two out of three parents rating it as “great” and “very helpful” or “extremely helpful.” All parents agreed or strongly agreed that the program helped them feel more empowered and equipped to protect their children from conflict, validated their experiences, provided useful tools, and offered strategies that felt doable, fitting, and easy to use. They also expressed strong approval of the program’s structure and content, all indicating they would recommend it to others. Parents particularly appreciated the mixed group sessions with other co-parents and the opportunity to shift their mindset, stating that the program helped them let go of trying to change their co-parent, which reduced their stress. The most appreciated parts of the program included offering better tools for managing difficult co-parent relationships and the coach-leading the sessions. Suggestions for improvement included making the program more affordable to increase access to the program for a wider range of families.

## DISCUSSION

Grounded on the premise that strengthening co-parenting fosters a healthier post-divorce environment for children, we evaluated BeH20 in two complementary samples. First, a large post-program survey of parents assessed perceived changes in their co-parenting relationship, ratings of program components, and overall acceptability of BeH20. Second, a multiple-baseline, single-case experimental design tracked daily shifts in animosity, respect, nonreactivity, and constructive vs. destructive conflict behaviors—both self-rated and as perceived in one’s co-parent—while also collecting open-ended feedback. Together, these studies provide preliminary evidence that BeH20 is perceived as helpful and engaging to parents and shows initial promise in reducing harmful co-parenting dynamics.

### Preliminary evidence of program-induced change in animosity

We found that participation in BeH20 was associated with gradual reductions in animosity and immediate increases in constructive conflict behaviors. Specifically, we observed a gradual reduction in angry, avoidant, and distrusting behaviors toward the co-parent and an immediate increase in calm discussions, support, and affectionate behaviors reported by parents, compared to their baseline levels of these same constructs. These changes are meaningful given prior research indicating that unresolved interparental conflict undermines effective co-parenting (Cronin et al., 2017) and contributes to child adjustment difficulties via heightened animosity (van Dijk et al., 2020). Theoretically, reduced animosity is also expected to lessen spillover effects that negatively impact parenting quality (Cox et al., 2001; Repetti, 1987). Establishing that BeH20 can engage these critical intervention targets is a critical step toward understanding whether and how BeH20 can support child adjustment after separation/divorce.

### Understanding change in constructive and destructive conflict behaviors

Constructive and destructive conflict behaviors changed, but not as expected. Destructive conflict declined prior to program onset and constructive conflict increased immediately post-baseline, with no further improvements during participation. This may reflect an awareness effect (Sedgwick & Greenwood, 2015), where court mandates or pre-treatment expectations lead to early behavior modification or biased responding to present oneself in a positive light. They may also be true effects, in which case the absence of sustained gains raises questions about optimal program dosage, pacing, and the need for booster sessions to support continued growth.

Further, when we adjusted for co-parent contact, intervention effects on constructive and destructive conflict were not significant. Instead, contact frequency was the strongest predictor, with more frequent contact associated with higher levels of both constructive and destructive conflict. This indicates that changes in conflict behaviors may be more closely linked to contact dynamics than program participation. Importantly, effective co-parenting is not defined by the frequency of contact, but by the intentionality and quality of communication. For example, our collective clinical experience suggests that some parents who report that less frequent but more intentional communication experience less conflict overall. Put simply, it is not how often parents communicate, but whether their interactions allow each parent to remain grounded and focused on their role as co-parents to support their child’s well-being. Future adaptations may benefit from tailoring strategies to different contact patterns (e.g., high vs. low frequency in contact), including supporting limited, purposeful communication when appropriate.

### Respect and nonreactivity: Resistance to change or in need of better measurement?

Measures of respect and nonreactivity remained largely unchanged. These findings echo prior work suggesting these constructs are deeply embedded in co-parenting histories and may be less amenable to brief intervention (Stolnicu et al., 2022). However, these findings warrant cautious interpretation given measurement limitations. The respect measure demonstrated low internal consistency (Cronbach’s *α* = 0.41), raising concerns about measurement reliability and sensitivity to change. In contrast, nonreactivity showed excellent reliability (Cronbach’s *α* = 0.94), providing greater confidence in the stability of those findings. These mixed results point to the need for more precise and conceptually aligned measurement approaches. For instance, respect may be more meaningfully captured by focusing on the *process* of respect rather than its *relational target*. Rather than measuring a generalized sense of respect in the co-parenting relationship, future measures could focus on respect as a behavior intentionally modeled in specific interactions with the co-parent—not because the co-parent earned the respect, but because the child deserves to witness respectful communication. Similarly, nonreactivity may be better understood not as the absence of emotional triggers but as the capacity to recognize activation, pause, and respond intentionally in alignment with parenting goals instead of simply reacting to one’s emotional experience in the moment. These reframed conceptualizations of respect and nonreactivity within a co-parenting context may not be fully captured through existing measures of the constructs more generally, underscoring the importance of integrating mixed-method research to refine measurement strategies to better capture the program’s putative change mechanism focused on value-driven, child-centered shifts in co-parenting behaviors.

### Underscoring the role of stakeholder engagement

Parents’ feedback on their experience in BeH20 offers insights into program acceptability, a key predictor of engagement and intervention effectiveness. Across two independent samples, parents consistently described BeH20 as helpful, empowering, and relevant to co-parenting challenges after separation. Parents highlighted the program’s practical guidance for managing conflict, separating marital dynamics from co-parenting responsibilities, and supporting their child’s relationship with both parents. Many parents reported feeling more equipped to handle difficult interactions and experienced a shift in mindset—moving away from efforts to change their co-parent and instead focusing on what they could control. Coach-led sessions and mixed-group discussions were valued for providing emotional support and actionable strategies. Suggestions to improve affordability further underscore the importance of developing scalable, cost-effective delivery models to broaden access for families.

This study provides preliminary evidence of target engagement, specifically whether the program successfully influences the proximal mechanisms it aims to change to benefit separated and divorcing families. Establishing these short-term effects is a necessary step to justify the resources required for larger-scale and longer-term evaluations to determine whether these benefits are sustained over time. This study also highlights the value of university and community partnerships in advancing practice-relevant research, especially for community-developed programs that were created outside of traditional research settings. Many programs, like BeH20, are developed in direct response to community needs and practitioner expertise but often lack formal evaluation. Collaborative partnerships provide an opportunity to systematically examine the effectiveness of these programs while preserving the elements that make them responsive and accessible to families. In this project, the community partner identified key areas of concern, ensuring the research addressed meaningful, practice-informed questions, while the university partner contributed methodological expertise to strengthen the quality of the evidence generated. The community and university partners collaboratively interpreted the results to better understand the nuances of the findings. This community-engaged approach enhances the rigor of program evaluation while supporting ongoing program refinement in ways that are grounded in real-world service delivery. As many interventions operate outside of academic settings, such partnerships are essential for building evidence for interventions that reflect the realities of community practice. Future work should continue to prioritize co-designed research that centers practitioner knowledge and community voice while also maintaining methodological rigor, together ensuring interventions remain both effective and responsive to the families they serve.

### Limitations

Findings from this pilot study are encouraging, but several limitations should be noted. First, we experienced considerable missing data, particularly on the daily and pre-post assessments. A common challenge in single-case designs is balancing the need for frequent assessments to capture patterns of change with participant burden. This design required at least five daily assessments before the program, which likely proved too demanding for many parents, considering the quantity of missing data. Additionally, only four parents completed both pre- and post-program assessments, limiting our ability to assess meaningful individual-level change in more macro constructs across the program period. This limits conclusions about the overall impact of the program. For future studies, alternative strategies, such as brief weekly check-ins integrated into sessions, may improve data completeness while maintaining measurement rigor. Feasibility of different assessment designs should be explored while considering practical constraints of the program providers, variability in enrollment timelines, and session start dates.

Second, internal consistency was low for the respect scale (*α* = 0.41) and only modest for animosity (*α* = 0.57), indicating substantial measurement error that likely undermined our ability to detect true change over time and may explain the null findings. Although we drew items from well-validated instruments, their reliability and sensitivity at the daily level remain untested. Future work should enhance reliability by refining item wording, evaluating alternative validated measures, or incorporating qualitative methods to more accurately capture day-to-day co-parenting dynamics.

Third, there was a miscommunication regarding the intervention’s intake process. The university partner initially understood intake to be assessment-only, but later learned it included some active intervention components (e.g., outlining processes to achieve co-parenting goals). This likely contributed to the observed pre-intervention improvements, including declines in destructive conflict during baseline and immediate increases in constructive conflict at intervention onset. Future partnerships should include thorough discussions, shadowing, or training on all program components from first contact through intake and session delivery to accurately map assessment timing with respect to intervention start time.

Fourth, this study examined only short-term pre–post effects, which limits understanding of whether observed benefits are sustained over time. However, it provides needed justification for resource allocation to future studies that include longer-term follow-ups to evaluate the sustainability or change of these initial improvements.

Future research should aim to reduce assessment burden, clarify program exposure points, account for real-world delivery constraints, and include long-term follow-ups to better capture how programs like BeH20 operate in real-world settings and sustain effects over time.

## CONCLUSION

This project investigated the effects of BeH20, a community-embedded co-parenting program, on separated and divorced parents for high levels of IPC. In a larger post-only survey (*N* = 101), parents showed a large pre–post improvement in perceived co-parenting quality (mean Δ = 1.99, SD = 1.76; Cohen’s *d* = 1.13), with 78% reporting positive change. Parents also rated core program components very positively (*M* = 3.99–4.56 on 5-point scales), expressed high overall satisfaction (*M* = 4.01), and indicated strong likelihood of recommending BeH20 to a friend (*M* = 4.02). In a rigorous multiple-baseline experiment, changes in daily intra- and interparental dynamics, including animosity, respect, nonreactivity, and conflict behaviors were evaluated for within-subject intervention effects. Together, findings offer preliminary evidence that BeH20 is perceived as acceptable and helpful by parents and can influence a key mechanism of change—decreasing animosity toward the other parent. Overall, the project illustrates the feasibility of applying rigorous experimental methods within a university–community partnership. Future research should continue these collaborations to explore additional mechanisms of change and to refine feasible data-collection processes for community-embedded program evaluations.

## Figures and Tables

**FIGURE 1:**
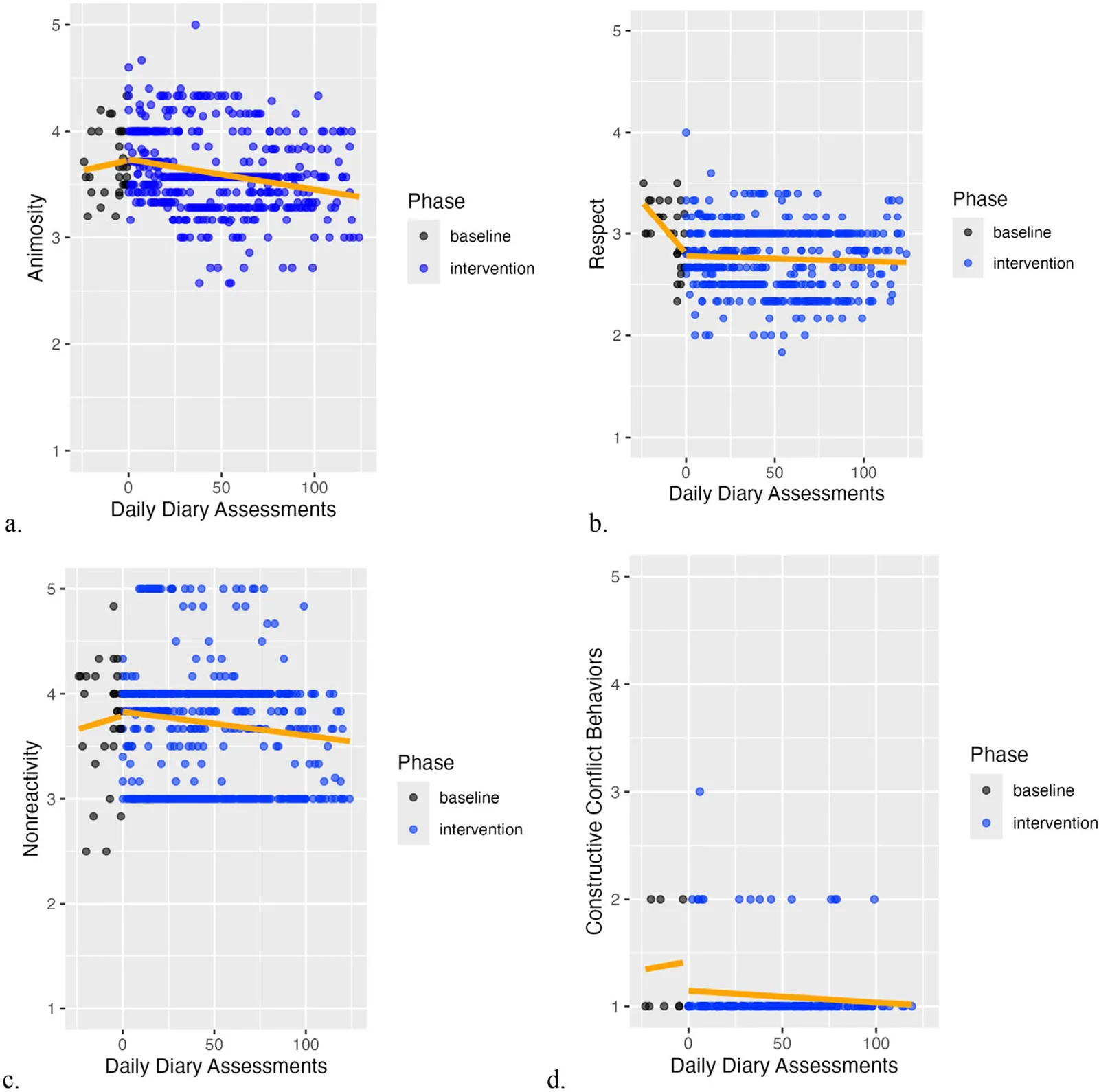

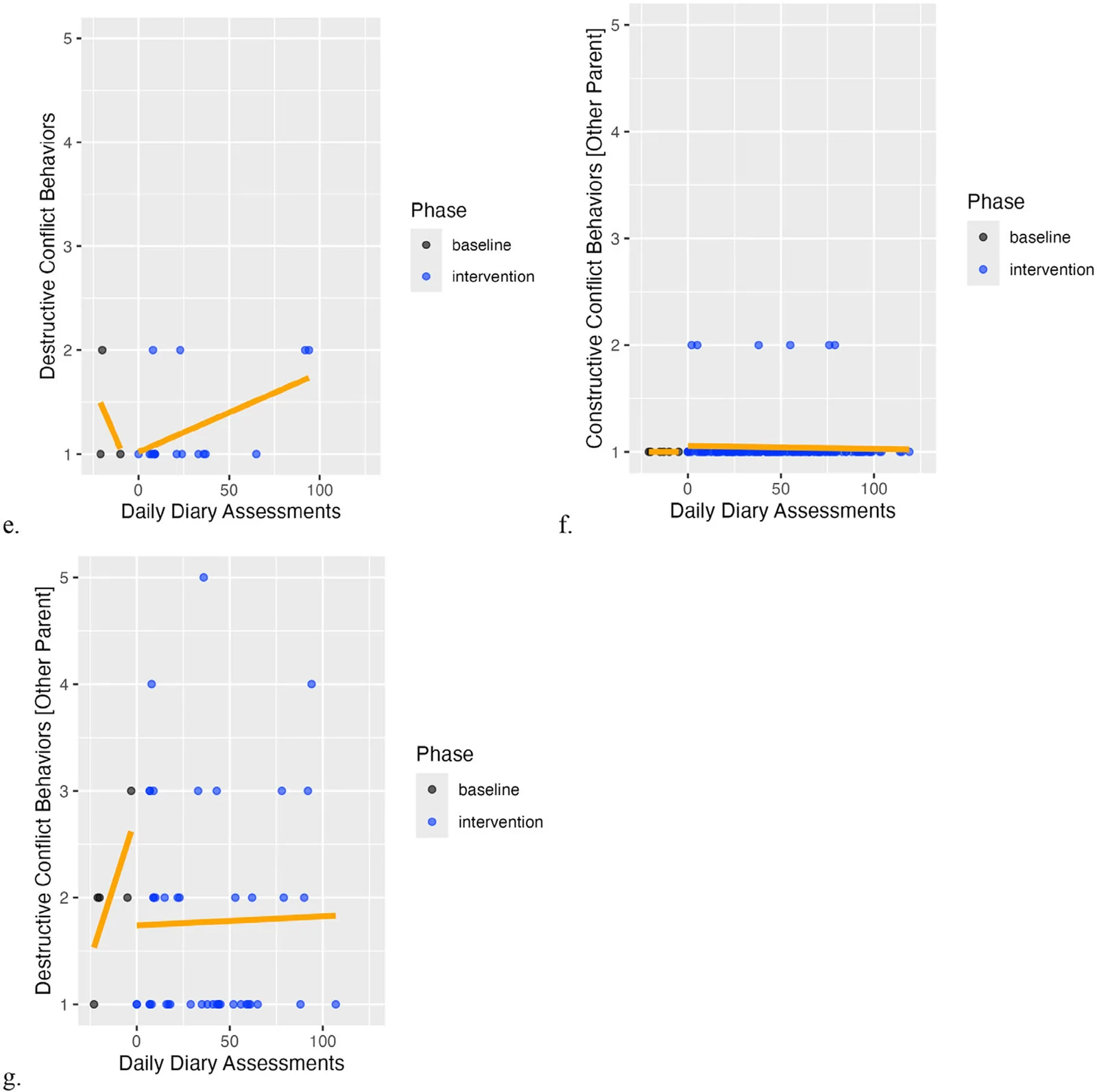
Graph depicting model-implied values of (a) animosity, (b) respect, (c) nonreactivity, (d) constructive conflict, (e) destructive conflict, (f) other parent’s constructive conflict, and (g) other parent’s deconstructive conflict scores across baseline and intervention phases.

**TABLE 1:** Unstandardized regression coefficients from mixed effects models assessing intervention effects on animosity, respect, nonreactivity, constructive conflict, and destructive conflict.

Effect Animosity	Model 1: Linear change		
	Estimate	SE	*p*-value
Intercept	3.69	0.12	<0.0001*
Phase	0.12	0.07	0.09
Time0 (baseline)	−0.01	0.01	0.16
Phase × Time0	0.01	0.01	0.39
Time0 (intervention)	−0.004	0.001	0.03*
Respect	Estimate	SE	*p*-value
Intercept	2.85	0.10	<0.0001*
Phase	−0.04	0.08	0.63
Time0 (baseline)	−0.01	0.01	0.11
Phase × Time0	0.01	0.01	0.15
Time0 (intervention)	−0.001	0.001	0.33
Nonreactivity	Estimate	SE	*p*-value
Intercept	3.61	0.17	<0.0001*
Phase	0.027	0.08	0.74
Time0 (baseline)	−0.007	0.01	0.52
Phase × Time0	0.02	0.01	0.11
Time0 (intervention)	0.01	0.01	0.18
Constructive	Estimate	SE	*p*-value
Intercept	0.04	0.14	0.75
Phase	0.39	0.13	0.003*
Time0 (baseline)	−0.02	0.01	0.17
Phase × Time0	0.02	0.01	0.21
Time0 (intervention)	−0.002	0.001	0.15
Destructive	Estimate	SE	*p*-value
Intercept	−0.03	0.07	0.66
Phase	0.14	0.07	0.06
Time0 (baseline)	−0.02	0.01	0.002*
Phase × Time0	0.02	0.01	0.002*
Time0 (intervention)	−0.001	0.001	0.31
Other parent constructive	Estimate	SE	*p*-value
Intercept	−0.02	0.12	0.85
Phase	0.31	0.11	0.007*
Time0 (baseline)	−0.01	0.01	0.62
Phase × Time0	0.00	0.01	0.65
Time0 (intervention)	−0.001	0.001	0.67
Other parent destructive	Estimate	SE	*p*-value
Intercept	0.09	0.15	0.54
Phase	0.15	0.15	0.33
Time0 (baseline)	−0.02	0.01	0.13
Phase × Time0	0.02	0.01	0.16
Time0 (intervention)	−0.001	0.001	0.12
Contact other parent and constructive	Estimate	SE	*p*-value
Intercept	−0.13	0.08	0.11
Phase	0.10	0.07	0.12
Time0 (baseline)	0.000	0.01	0.98
Phase × Time0	0.000	0.01	0.95
Contact other parent	1.00	0.03	<0.0001*
Time0 (intervention)	0.001	0.001	0.59
Contact other parent and destructive	Estimate	SE	*p*-value
Intercept	−0.01	0.07	0.92
Phase	0.07	0.07	0.26
Time0 (baseline)	−0.009	0.01	0.11
Phase × Time0	0.009	0.01	0.12
Contact other parent	0.12	0.02	<0.0001*
Time0 (intervention)	−0.0002	0.0004	0.56

*Note*: Effect interpretations are as follows: Intercept: baseline phase intercept, Phase: immediate shift in level between baseline and intervention phases, Time0 (baseline): baseline phase linear slope, Time (intervention): intervention phase linear slope, Phase × Time0: change in linear slope between baseline and intervention phases.

Abbreviations: Estimate, unstandardized regression coefficient; SE, standard error.

* *p* < 0.05.

## Key points for the family court community

- High-conflict divorce and separation create significant challenges for co-parenting, underscoring the need for effective, accessible interventions.
- BeH2O^®^ is a community-based co-parenting program that teaches practical communication skills, conflict-resolution strategies, and ways to support children’s relationships with both parents.
- Parents reported improvements in co-parenting quality and expressed high levels of satisfaction with the program.
- Daily assessment data showed a significant reduction in co-parental animosity during participation, identifying a key mechanism through which the program may improve co-parenting relationships.
- Findings provide preliminary evidence that community-embedded co-parenting programs can be effectively evaluated through university–community partnerships and may offer a scalable approach to supporting high-conflict families.
